# Non-contact acquisition of brain function using a time-extracted compact camera

**DOI:** 10.1038/s41598-019-54458-7

**Published:** 2019-11-28

**Authors:** Takamasa Ando, Tatsuya Nakamura, Toshiya Fujii, Teruhiro Shiono, Tasuku Nakamura, Masato Suzuki, Naomi Anzue-Satoi, Kenji Narumi, Hisashi Watanabe, Tsuguhiro Korenaga, Eiji Okada, Yasunori Inoue

**Affiliations:** 1 Technology Innovation Division, Panasonic Corporation, Moriguchi, Osaka 570-8501 Japan; 2 Department of Electronics and Electrical Engineering, Keio University, Yokohama, Kanagawa 223-8522 Japan

**Keywords:** Optical sensors, Imaging and sensing

## Abstract

A revolution in functional brain imaging techniques is in progress in the field of neurosciences. Optical imaging techniques, such as high-density diffuse optical tomography (HD-DOT), in which source-detector pairs of probes are placed on subjects’ heads, provide better portability than conventional functional magnetic resonance imaging (fMRI) equipment. However, these techniques remain costly and can only acquire images at up to a few measurements per square centimetre, even when multiple detector probes are employed. In this study, we demonstrate functional brain imaging using a compact and affordable setup that employs nanosecond-order pulsed ordinary laser diodes and a time-extracted image sensor with superimposition capture of scattered components. Our technique can simply and easily attain a high density of measurement points without requiring probes to be attached, and can directly capture two-dimensional functional brain images. We have demonstrated brain activity imaging using a phantom that mimics the optical properties of an adult human head, and with a human subject, have measured cognitive brain activation while the subject is solving simple arithmetical tasks.

## Introduction

Techniques for imaging in the presence of scattering media are crucial to the fields of biometrics and biomedical neuroscience. Near-infrared spectroscopy (NIRS)^1,2^ uses source–detector probes located far apart when monitoring blood haemoglobin levels in the cerebral cortex to enhance the detection of deep component signals. Researchers at NIRS have recently developed a high-density diffuse optical tomography (HD-DOT) technique^3–8^ to improve spatial resolution by using multiple probes with additional short source–detector (SD) distances. HD-DOT can image quantitative^8,9^ haemoglobin concentration changes three-dimensionally by solving the inverse problem with spatial sensitivity distribution. Time-domain functional near-infrared spectroscopy (TD-fNIRS)^10–14^ can be used to monitor tissue oxygenation using a picosecond ultrashort pulse laser and a fast-gated single-photon avalanche diode (SPAD) for contactless detection. Two-dimensional image acquisition has also been demonstrated by scanning^11^ the illumination spot. Time-gated measurements using an intensified CCD camera have also been adopted to detect absorption changes in deep tissues^15–23^, and two-dimensional imaging^22^, and *in vivo* experiments^23^ have also been performed.

In our work using a separated-light contactless extraction (SLICE) camera, we have directly acquired brain functional images employing a compact and inexpensive setup that uses ordinary nanosecond laser diodes and a compact camera’s image sensor without an image intensifier. In this paper, we demonstrate brain activity imaging using a phantom that mimics a human head and present *in vivo* measurements of task-evoked neural activity obtained in an adult human subject. In our tests, we employed nanosecond short-pulse illumination with a flat-top intensity distribution to directly obtain two-dimensional images. Since our image sensor-based technique eliminates the physical limitations on spatial resolution that are a function of the SD distance, higher-density measurements were achieved. Our technique, which does not require a scanning process, can effect zero time-delayed bounded capture of an extensive cerebral blood-oxygenated area. The use of ordinary laser diodes and an image sensor obviates the need to attach and align numerous probes to the subject.

## Results and Discussion

### Principle of time-extracted non-contact imaging

**Figure 1:**
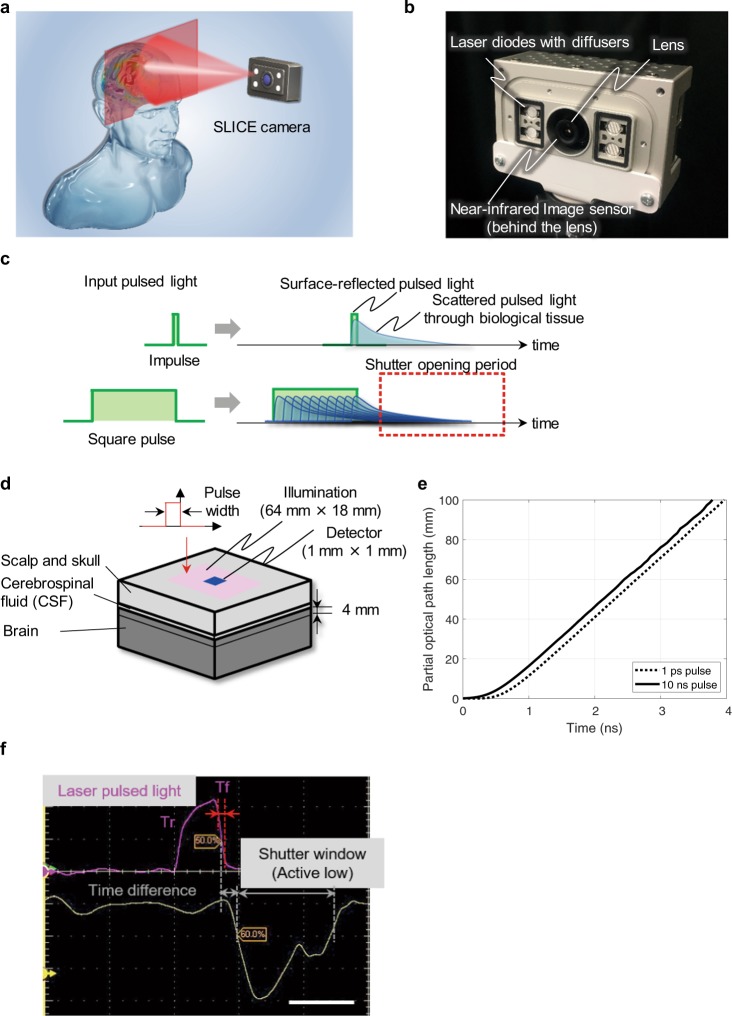
Time-extracted non-contact imaging. (**a**) Schematic of brain blood flow captured with the zero-contact SLICE camera. (**b**) Component parts of the camera. (**c**) Principle of capturing deep tissue images with a compact camera using ordinary laser diodes. (**d**) Modelling of light propagation in a simplified head model to analyse the influence of the pulse width of the input illumination. The size of the illuminated region is 64 × 18 mm. The detector, measuring 1 × 1 mm, is located in the centre of the illuminated region. Both were placed on the top surface of the phantom. The thickness and optical properties of each layer of the phantom are shown in Table 1. The mean optical path length of propagated light which passes the brain layer between 0–4 mm in depth was computed using a Monte Carlo simulation. (**e**) Relationship between arrival time of the returned light to the detector and mean partial optical path length. 0 ns on the time axis indicates that the pulsed light was turned off. Pulse widths of the input illumination were 1 ps and 10 ns. (**f**) Waveform of a laser pulsed light and the shutter driving signal of the SLICE camera, measured using a two-channel oscilloscope (Tektronix DPO 7254). Laser-pulsed light was detected with a photo detector and transformed from light intensity to a voltage level. Scale bars: 10 ns.

Our proposed method can capture the two-dimensional distribution of brain blood flow changes using a non-contact, compact setup that employs image sensor-based capture (Fig. 1a and Supplementary video). As shown in Fig. 1b, the SLICE camera was realised using a camera module equipped with two 750-nm laser diode (LD) sources (GH0752WA2G, manufactured by Sharp) and two 855-nm LD sources (22007682, manufactured by JDSU) that emit nanosecond square-pulsed light, and an image sensor^24^ with an 11-ns shutter time-window for detecting near-infrared light, manufactured by Panasonic Semiconductor Solutions Co., Ltd. The pixel structure of the image sensor consists of silicon-based stacked deep photodiodes that enhance the quantum efficiency at a wavelength of 850 nm^24^. The shutter timing of the image sensor is controlled within the camera by synchronizing it with the timing at which the LDs emits pulsed light. Flat-top diffusers are attached to the front of the laser diodes to illuminate the objects uniformly. The diffusers shaped the laser beams into a square pattern and the spatial intensity distribution was flat within this pattern.

Figure 1c shows the input pulsed light, the pulsed light reflected from the surface, and the scattered pulsed light after it passes through the biological tissue. Since the scattered pulsed light is a combination of light beams that have travelled along various optical paths, the scattered pulsed light has a “tail” at the trailing edge of the pulsed light. This is because the time delay is longer than that of the surface reflection component. The ratio of input light that reaches deep tissues tends to increase probabilistically as a function of the optical path length of the scattered light. To efficiently extract the late-arriving scattered components, which include information pertaining to the deep tissues, the electronic shutter is opened only at a point after the falling edge of the surface-reflected pulsed light has arrived at the image sensor (Dotted square in red in Fig. 1c).

Time-resolved detection can be applied to both the impulse, which is performed conventionally, and square-pulsed light. The optical path length of the pulsed illumination for input light passing through a simplified head model (Fig. 1d) was calculated by analysing the propagation of light using a Monte Carlo simulation. The number of photons for the calculation was 10 billion. Figure 1e shows mean partial effective optical path length, which was propagated between 4 mm in depth from the brain surface, of the returning light entering the detector. The partial optical path length with input light from a 10-ns pulse width was equivalent to or longer than the 1-ps pulse width. This result means that the longer the optical path length through a biological tissue, the greater the quantity of information on deep tissue components is carried by that light. Our results indicate that square-pulsed light can be used to detect partial optical path length at deep layers equally as well as impulse light, demonstrating that low-cost ordinary laser diodes with wide pulse widths, which also have the benefit of being compact, are sufficient to distinguish between superficial and deep components. Furthermore, long-optical-path components can be enhanced by superimposition (Fig. 1c) when square-pulsed light is input.

Here, we explain the effect of superimposition by comparing the detected intensity between the impulse-input and square-pulsed-input using Monte Carlo simulation, then show the calculation of the generated electrons on an image sensor. The simulation results in Supplementary Fig. S1 show detected intensity by the sensor with an 11-ns time window. The pulse widths of the input light were 10 ps and 11 ns, and the input intensity was 1 (a.u.) per 10-ps pulse-width. The sensor was placed on the surface of the object model in this simulation, and 0 ns in Supplementary Fig. S1 shows the timing when the end of the input light is reflected on the surface. When the shutter opened with an 0.8-ns delay after the end of the surface reflection, as shown in Supplementary Fig. S1, the detected light intensity of the nanosecond-square-pulse (11 ns) was almost three orders of magnitude greater than that with the impulse (10 ps) response. This was the effect of integration of the trailing edge. The ratio of the detected light intensity to input light intensity per unit area was increased to 1: 2.5 × 10^−6^.

The number of generated electrons on an image sensor in this system can be calculated using the propagation result of the detected light in the previous paragraph. Light intensity per frame on a pixel of the image sensor *I*_s_(*t*), where *t* is the shutter timing delay in Supplementary Fig. S1, can be written as (see Supplementary Fig. S2):

1 $${I}_{s}(t)=P\times n\times S\times R(t)\times \varOmega /2\pi \times a$$

where *P* is the light intensity of a single-input-pulse per unit area on the object surface, *n* is the number of light sources, *S* is the projected area of 1 pixel on the object surface, *R*(t) is the reflection ratio of light that passed through and scattered within the object, *Ω* is the solid angle from the object point to the aperture of the lens, and *a* is the number of captured pulses per frame.

When the pulse-width of the input light was 11 ns at a wavelength of 850 nm, *P* was 4.8 × 10^−7^ mJ at *ϕ*7 mm at distance *L* = 100 mm. The number of light sources *n* attached to the SLICE camera was two, and *S* was 0.07 × 0.07 mm. The reflection ratio *R*(t), when the shutter timing delay *t* = 0.8 ns, was 2.5 × 10^−6^. The aperture *D* of the lens was *ϕ*4 mm, the solid angle *Ω* was 1.3 × 10^−3^ sr and *a* was 440,000 times. Light intensity *I*_s_ in Eq. (1) was therefore 2.7 × 10^−17^ J, so when the sensitivity of the silicon photodiode was 0.3 A/W, the number of generated electrons was 51 e^−^. On the other hand, the noise in this system consisted of fixed patterns and random noise such as dark current and shot noise, respectively. The dark current generated only a few electrons, since the total opening time of electric shutter was 4.8 ms per frame^24^. Shot noise, which is the main noise on our camera, is equal to the square root of the generated electrons on the sensor and was 7 electrons. Spatial and temporal low pass filtering can be applied to the acquired images to improve the SN, since the standard width of the sulcus, which is the functional unit in the human cerebral cortex^25,26^, is 10 mm in spatial resolution, and the typical haemodynamic to neural activation consists of gradual reaction in the order of seconds^27^. Fixed pattern noise was also removed using subtraction from the captured signal by detection without illumination by the LDs during the same frame.

The fall time (Tf) characteristic of the output light from laser diodes is a contributory factor to the detection of changes in haemoglobin in the cerebral cortex. We have designed a circuit board using enhancement mode gallium-nitride-on-silicon field effect transistors (EPC2040, EPC Corporation), which output the driving current of the laser diodes. Their switching characteristic is fast for their low gate charge: they can force the Tf of the output light to fall steeply within 1 nanosecond (at 10–90%) as shown in Fig. 1f. The time increases from left to right in Fig. 1f. The electronic shutter in this system is realised using a time-extracted control method that alternates between accumulating electric charge on floating diffusion (FD) layers and discharging that charge through the drain^28^.

### Verification using a biological tissue-equivalent phantom

**Figure 2:**
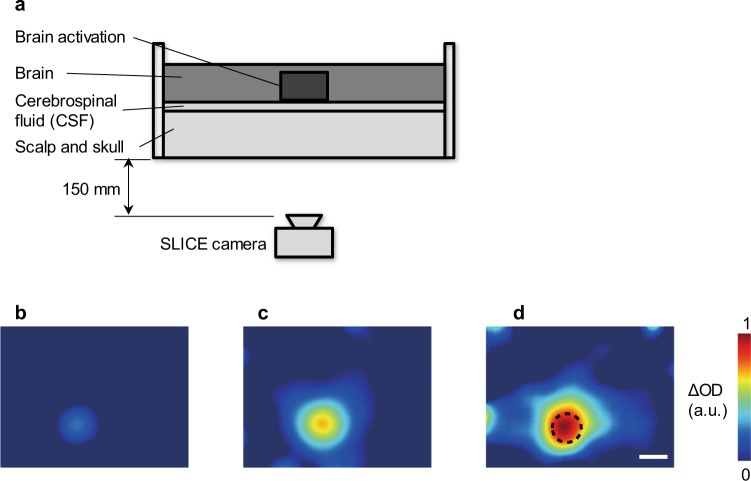
Imaging through a biological tissue-equivalent phantom using a SLICE camera. (**a**) Experimental setup for brain activity imaging with the SLICE camera. The head phantom consists of three parts: the superficial tissue (scalp and skull), cerebrospinal fluid (CSF), and brain. The thickness and optical properties of these parts are shown in Table 1. The bottom wall consists of two layers that model the superficial tissue and the CSF. The superficial tissue was constructed of epoxy resin containing TiO_2_ to alter the scattering coefficient and near-infrared absorbing dye to alter the absorption coefficient. The CSF layer was made of epoxy resin containing TiO_2_. The phantom comprised a water bath filled with intralipid solution and black ink to simulate brain tissue. An absorption rod was inserted into the intralipid solution to mimic the absorption changes caused by brain activation. The rod was made of epoxy resin with TiO_2_ and near-infrared absorbing dye, as was the bottom part of the phantom. The diameter of the absorption rod was 10 mm. (**b**–**d**) Images captured by the SLICE camera. The shutter starting times in (**b**–**d**) are respectively 0, 400, and 800 ps from the arrival time at half maximum of the end of the pulse. The values in (**b**–**d**) are shown as ∆OD, which represents the change in the intensity of the detected light compared to the initial data acquired without brain activation. Each image in (**b**–**d**) is normalized by the maximum value in (**d**). The dotted circle in (**d**) indicates the position of the absorption rod. Scale bars: 10 mm.

The biological tissue-equivalent phantom^29–32^ employed to demonstrate the principles of the proposed system is shown in Fig. 2a. It consists of superficial tissue (scalp^33^ and skull^34^), cerebrospinal fluid^29^ (CSF) and brain^35^ layers that mimic the optical properties of an adult human head^29,33–35^. The thickness and optical properties of each layer are shown in Table 1.

**Table 1:** Thickness and optical properties of the layers of the head model.

	Thickness (mm)	Transport scattering coefficient (mm^−1^)	Absorption coefficient (mm^−1^)
Scalp and skull	10	1.8	0.019
Cerebrospinal fluid	2	0.3	0
Brain	∞	2.1	0.04

Figure 2b–d shows images captured by the SLICE camera. The values in the images represent ∆OD^29^, the change in the intensity of the detected light from the initial state without brain activation. This result reveals that our SLICE camera can acquire two-dimensional changes in haemoglobin in deep tissue as light-signal changes. The later the shutter starting time, the stronger the ∆OD of the deep tissues became, as shown in Fig. 2b–d. When the shutter opened later than in Fig. 2d, the ∆OD images were swamped by noise, since the detected light had become too faint. The optimal shutter opening timing is therefore set in consideration of the amount of noise. This result also indicates that our method, which detects deep tissues with high density, can capture images without mislocalisation or loss of information.

The following two steps are the key to realizing high quality images of changes in haemoglobin in deep tissue in scattering media. The first step is to acquire measurement data with a high density of sampling points, and the second step is to correct the spatial light scattering from measurement data by calculation. The quality of the images in the second step depends on the measurement data acquired in the first step.

**Figure 3:**
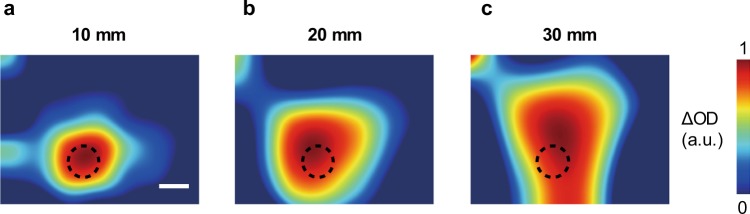
Effect of the number of sampling points on the detected images. (**a**–**c**) Binning images formed from the image in Fig. 2d. Binning pitches are respectively 10, 20, and 30 mm in (**a**–**c**). Bicubic interpolation with the same pixel count as the original image was applied after the binning process in (**a**–**c**). Colour scales of ∆OD were normalized by the maximum value in each image. The scale bar is the same as in Fig. 2d.

Conventional fNIRS detects deep tissue using a spatial separation method in which the sources and detectors are 30 mm distant from each other. HD-DOT, which has a sampling pitch of 10 mm, also uses source-detector pairs at a distance of 30 mm for efficient acquisition of deep tissue information. This spatial separation method gathers information from a broad area in the deep layers over the 30-mm width. Figure 3 shows the dependency of sampling pitch of the detected images. Images in Fig. 3a–c were formed by binning the image, which assumed the spatial separation method, in Fig. 2d with sampling pitches of 10, 20, and 30 mm, respectively. After the binning process, the images were interpolated to the same pixel counts as the original version. The process in Fig. 3a–c assumes measurement using conventional fNIRS, which has a sparse configuration of source and detector probes. With the 30-mm sampling pitch in Fig. 3c, the absorption rod was illustrated as being larger and in the wrong position because of too few sampling points (Supplementary Figs. 3–6 also shows an experimental comparison with conventional fNIRS). Although the distribution of brain activation is better detected with a higher number of sampling points, the sampling density in Fig. 3a shows the physical limitations of the conventional method that uses physical probes, compared to the images taken by our method using a camera that takes 1,600 measurements/cm^2^, shown in Fig. 2d.

The second step, which is a common problem with fNIRS measurements, was not executed in this study; however, conventional reconstruction processes^7,36–39^ such as those for HD-DOT, are also applicable to our measurement data.

### *In-vivo* testing

**Figure 4:**
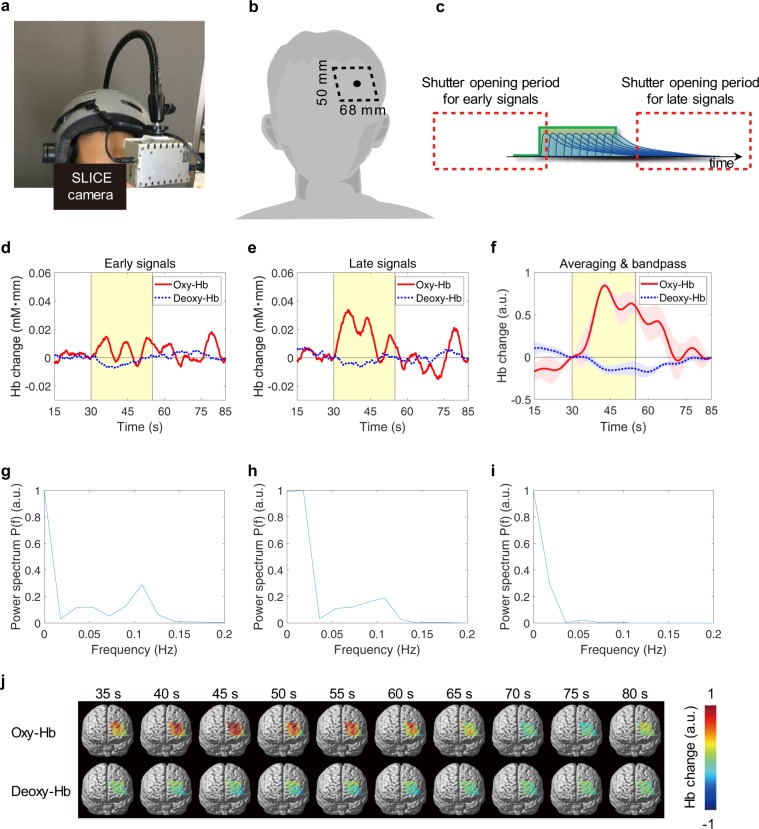
Changes in haemoglobin in a human subject acquired using a SLICE camera during an arithmetic task. (**a**) Setup for the experiment. The camera was held at a distance of 7 cm from the subject’s forehead by an arm attached to the helmet. (**b**) Field of view of the camera on the subject. (**c**) Shutter opening timing in the experiment. (**d**–**f**) Cortical responses to cognitive stimuli. Solid and dotted lines show the changes in oxy and deoxyhaemoglobin, respectively. The signals in (**d**,**e**) were captured using the early and late parts, respectively, of an arriving pulse. The result in (**f**) was taken with late shutter timing and subjected to 5-trial averaging and a band pass of between 0.01 and 0.1 Hz. To reduce the biasing influence of higher signals, the averaging was executed after the values in each trial had been normalized by the maximum change in oxyhaemoglobin in each trial. Shadings indicate standard errors. The yellow background in (**d**–**f**) refers to periods during the task. The solid circle in (**b**) refers to a selected pixel plotted in (**d**–**f**). (**g**–**i**) Power spectra of the haemoglobin signals in (**d**–**f**), respectively. Each of the power spectra in (**g**–**i**) was normalized by the maximum value. (**j**) Two-dimensional captured images of changes in oxy- and deoxyhaemoglobin from 35–80 s in (**f**). A spatial Gaussian filter with 10 pixels as standard deviation was applied.

A demonstration with a human subject to measure cognitive brain activation while solving simple arithmetical tasks in combination with 2-back tasks is shown in Fig. 4, which illustrates the response by our camera to the subject’s blood oxygen levels. During the task, problems comprising single-digit additions were displayed randomly in sequence on a monitor. The subject was instructed to calculate the sums of two numbers, memorize them sequentially and mentally answer the problem two steps earlier. Light components with wavelengths of 750 and 855 nm were alternately captured in odd and even row pixel lines every 4,000 pulses to record changes in oxy- and deoxy-haemoglobin^40^. This operation was performed 55 times per frame to reduce the time lag between the two wavelengths. Our technique can capture information on both deep and superficial tissue by using time differential capture of the early and late components of the arrival pulses, as shown in Fig. 4c. The electric shutter for late signals opened with the timing required for efficiently detecting deep tissues in the head phantom, such as in Fig. 2d. The early-arriving signals were captured by opening the shutter window much earlier than the time-scattered pulsed light arrived at the image sensor, and closing it 1 ns after the leading edge arrived, since the shutter window was 11 ns. The leading edge of the scattered pulsed light was therefore detected for 1 ns; as a result, it included not only surface-reflected light but also a proportion of internally-scattered components from under the surface, such as the scalp layer.

Figure 4d–f shows the results of *in vivo* testing. In the test, the subject rested for 30 s, performed the task for 25 s, then rested for 30 s. The solid circle in Fig. 4b refers to a selected pixel plotted in Fig. 4d–f, which is located around Brodmann area 46 and is related to working memory^41,42^. Oxyhaemoglobin data of late signals in Fig. 4e can identify task initiation. However, task-related activation was not observed with early signals in Fig. 4d, which includes mainly the skin blood component on the superficial layer. Figure 4g,h show the power spectra of Fig. 4d,e. Low-frequency oscillations (LFOs) around the 0.1 Hz component were detected, such as Mayer waves^43,44^, which derive from the baroreflex in the systemic regulation of the cardiovascular system. They appear to be caused by changes in haemoglobin in the superficial layer, since similar temporal patterns were seen in both Fig. 4d,e. This result indicates that two-dimensional changes in haemoglobin in only the cerebral component can be extracted by comparison of these two components in Fig. 4d,e, although late signals that pass through both deep and shallow areas can include superficial changes in haemoglobin. Although not shown here, the spectrum around 1.1 Hz was also faintly detected and appears to be derived from the heartbeat.

The periodic noise in Fig. 4e, which appears to be caused by superficial tissue, can be attenuated by averaging and using a band pass filter as shown in Fig. 4f. The result in Fig. 4f, which was captured with late shutter timing, was applied with an averaging of five trials and a band pass of between 0.01 and 0.1 Hz which included the low frequency of the task and rest periods. The averages and standard errors of the changes in oxy- and deoxyhaemoglobin are plotted as solid lines and shading, respectively, in Fig. 4f. As shown in Fig. 4f, the periodic component was decreased and the task-related signals were obtained more distinctly. LFOs around 0.1 Hz in Fig. 4i were also reduced by the averaging and band pass method. The two-dimensional distributions of changes in haemoglobin in Fig. 4f are shown in Fig. 4j. Most visible changes in oxyhaemoglobin in the image are found in the upper left and middle regions, which are around Brodmann areas 9 and 46.

## Conclusion

We have demonstrated two-dimensional single-shot direct captures of deep tissue using square-pulsed illumination and a time-extracted digital compact camera employing a non-contact configuration. This technique realized a high number of measurement points on the image without mislocalisation or loss of information. The results acquired in this study were optical topography images that were not subjected to any reconstruction processes. However, they could be converted to DOT images by estimating the spatial sensitivity distributions and reconstructing the inverse problem. Any drawbacks to the reconstruction process could be mitigated by the high density of points.

Although a SLICE camera cannot work through the hair on a subject’s head, it can detect the frontal lobes, which are associated with the higher brain functions unique to humans. However, it is important to note that imaging moving objects remains a challenge. This drawback might be solved by using the image-based motion stabilization mechanism of consumer digital cameras, since our camera can also capture textural images of objects.

Our device eliminates certain limitations to conventional systems, such as system size, cost and the probe alignment requirement of conventional functional brain imaging systems, making our device of greater practical use. For these reasons, we anticipate that this camera will revolutionize sensing in the neuroimaging field and pave the way towards the evolution of human-robot interactions.

## Methods

### Monte carlo simulation

Light propagation in the head model in Fig. 1e and Supplementary Fig. S1 was analysed by Monte Carlo simulation. Illumination light was input to the surface of the phantom. This analysis recorded total optical path lengths from the input point to the exit point under ray tracing, as well as partial optical path lengths which passed the brain layer between 0–4 mm in depth. Those with mean partial optical path length are plotted in Fig. 1e. 0 ns on the time axis indicates that the pulsed light was turned off.

In Supplementary Fig. S1, the shutter timing dependency of detected scattered light is resolved by recording the time of propagation and the number of rays that returned to the detector measuring 10 × 10 mm. Assumed intensity of the rays during an 11-ns time-window is plotted in Supplementary Fig. S1.

### Phantom evaluation

The phantom comprised a water bath filled with intralipid solution and black ink to model brain tissue. A cylindrical absorption rod with a diameter of 10 mm was inserted into the intralipid solution to model the changes in haemoglobin caused by brain activation. The transport scattering and absorption coefficients were 2.1 and 0.05 mm^−1^, respectively.

The developed camera was positioned 150 mm from the surface of the phantom (Fig. 2a). To ensure stable light emission, the experiments were executed 60 minutes after switching on the LDs. There were 440,000 emitted pulses and shutter openings per frame. The frame rate was 5 fps and the field of view was 64 × 44 mm. The effective pixel count on the image sensor was 320 × 240, so about 2,700 measurements/cm^2^ were acquired. The data captured at a wavelength of 855 nm was used in the evaluation. Background noise such as ambient light was subtracted from the captured signal by detection with different FD layers on the image sensor without illumination from the LDs during the same frame. Dark current noise on the sensor was also removed using this process.

The camera first captured a phantom image from the direction of the scalp and skull layer without the absorption rod in the brain water bath to model the rest state in which the brain was not activated. Secondly, the absorption rod was inserted into the water bath to model brain activation, and the change in intensity from the initial rest image to the activated image was evaluated to verify the detection of deep tissue.

Three differentiated images in Fig. 2b–d were captured as independent measurements. The values shown in Fig. 2b–d were computed as ∆OD (=−∆ln*I*, where *I* is the intensity of the detected light), which was the change in the intensity of the detected light from the initial state, taken with only the brain component, to an active state taken with the absorption rod in the brain. A spatial Gaussian filter with 18 pixels as standard deviation was applied.

### Adult head measurement

The response of the blood oxygen levels of the subject during a 2-back task on a monitor consisting of the addition of one order of magnitude was measured using our camera. Light components with wavelengths of 750 and 855 nm were alternately captured in odd and even row pixel lines every 4,000 pulses to record changes in oxy- and deoxy-haemoglobin^35^. This operation was performed 55 times per frame to reduce the time lag between the two wavelengths.

The image sensor in this work includes multiple FD layers per photodiode in each pixel, so that two types of light components can accumulate in each FD layer. In addition to standard mode, in which the camera captured only the late-arriving signals, early-arriving signals were detected using another FD layer on the sensor in the same frame by alternately selecting the FD layer to collect the electrons from the photodiode, as shown in Fig. 4d,e. FD layers to accumulate the electrons were replaced every 4000 pulses in each frame to reduce the time lag between early- and late-arriving signal detection. The detected optical density data from the camera were converted into changes in haemoglobin, which were the changes in the product of haemoglobin and optical path length. Ambient light components, such as from the fluorescent lighting in the laboratory, were also captured with a different FD layer with the laser diodes off in the same frame to remove them from the captured signal, using the same technique as in the previous phantom experiment. The frame rate of the camera was 5 fps. The SLICE camera was held by a flexible arm that was attached to a helmet worn by the subject to minimize the influence of his body movements. The camera was located on the left side of the prefrontal cortex, as shown in Fig. 4a. The distance between the forehead of the subject and the camera was 7 cm. The location of the detected area on the head of the subject was determined based on the international 10–10 system, and the field of view of the camera, which used a wide-angle lens, was a 68 × 50 mm area on the subject’s forehead (Fig. 4b).

A healthy male volunteer in his mid-twenties participated in the following measurements after providing his informed consent, having been given a full description of the study beforehand. During the test, the subject rested for 30 s, performed the task for 25 s, and then rested for 30 s. A baseline correction was applied to the time series data representing the changes in haemoglobin at 30- and 85-s intervals. A spatial Gaussian filter with 10 pixels, which is 2 mm on the subject’s forehead, as standard deviation and a temporal low pass filter between 0.01 and 0.1 Hz were applied to reduce shot noise. The right and low edges of the detected images were removed from the data on the plot in Fig. 4j, since part of the subject’s hair and eyebrow intruded slightly into the camera’s field of view.

### Ethics

The experiment was conducted in a laboratory at Panasonic Corporation. This study was approved by Panasonic Corporation’s Ethical Review Board and followed the Helsinki Declaration and guidelines of the Finnish Committee for Research Ethics. All the study participants were briefed on the purpose of the study, and written informed consent was obtained from them before the experiments. The study participant also gave his informed consent to publication of the image of the experimental setup shown in Fig. 4a.

## Supplementary information

Supplementary information

Concept video

## References

[1] Gibson AP, Hebden JC, Arridge SR (2005). Recent advances in diffuse optical imaging. Phys. Med. Biol..

[2] Strangman G, Culver JP, Thompson JH, Boas DA (2002). A quantitative comparison of simultaneous BOLD fMRI and NIRS recordings during functional brain activation. NeuroImage.

[3] Eggebrecht AT (2012). A quantitative spatial comparison of high-density diffuse optical tomography and fMRI cortical mapping. NeuroImage.

[4] Eggebrecht AT (2014). Mapping distributed brain function and networks with diffuse optical tomography. Nature Photon..

[5] Zeff BW, White BR, Dehghani H, Schlaggar BL, Culver JP (2007). Retinotopic mapping of adult human visual cortex with high-density diffuse optical tomography. Proc. Natl. Acad. Sci. USA.

[6] Joseph DK, Huppert TJ, Franceschini MA, Boas DA (2006). Diffuse optical tomography system to image brain activation with improved spatial resolution and validation with functional magnetic resonance imaging. Appl. Opt..

[7] Yamashita O (2016). Multi-subject and multi-task experimental validation of the hierarchical Bayesian diffuse optical tomography algorithm. NeuroImage.

[8] White BR, Culver JP (2010). Quantitative evaluation of high-density diffuse optical tomography: *in vivo* resolution and mapping performance. J. biomed. opt..

[9] Zhan Y, Eggebrecht A, Dehghani H, Culver J (2011). Quantitative evaluation of systematic imaging error due to uncertainty in tissue optical properties in high-density diffuse optical tomography. Proc. SPIE.

[10] Mazurenka M (2012). Non-contact time-resolved diffuse reflectance imaging at null source-detector separation. Opt. Express.

[11] Torricelli A (2014). Time domain functional NIRS imaging for human brain mapping. NeuroImage.

[12] Zucchelli L, Contini D, Re R, Torricelli A, Spinelli L (2013). Method for the discrimination of superficial and deep absorption variations by time domain fNIRS. Biomed. Opt. Express.

[13] Wabnitz H, Möller M, Liebert A, Walter A, Macdonald R (2005). A time-domain NIR brain imager applied in functional stimulation experiments. Proc. SPIE.

[14] Wabnitz H (2010). Time-resolved near-infrared spectroscopy and imaging of the adult human brain. Adv. Exp. Med. Biol..

[15] Niedre MJ (2008). Early photon tomography allows fluorescence detection of lung carcinomas and disease progression in mice *in vivo*. Proc. Natl. Acad. Sci. USA.

[16] Zhao Q (2011). Functional tomography using a time-gated ICCD camera. Biomed. Opt. Express.

[17] Joseph DK, Boas DA (2018). Time-gated optical system for depth-resolved functional brain imaging. J. biomed. opt..

[18] Sawosz P, Kacprzak M, Liebert A, Maniewski R (2007). Application of time-gated, intensified CCD camera for imaging of absorption changes in non-homogenous medium. IFMBE Proceedings.

[19] Selb J, Stott JJ, Franceschini MA, Sorensen AG, Boas DA (2005). Improved sensitivity to cerebral hemodynamics during brain activation with a time-gated optical system: analytical model and experimental validation. J. biomed. opt..

[20] Selb J, Joseph DK, Boas DA (2006). Time-gated optical system for depth-resolved functional brain imaging. J. biomed. opt..

[21] Selb J, Dale AM, Boas D (2007). Linear 3D reconstruction of time-domain diffuse optical imaging differential data: improved depth localization and lateral resolution. Opt. Express.

[22] Poulet P (2013). A time-gated near-infrared spectroscopic imaging device for clinical applications. Proc. SPIE.

[23] Lange F, Peyrin F, Montcel B (2018). (2018). Broadband time-resolved multi-channel functional near-infrared spectroscopy system to monitor *in vivo* physiological changes of human brain activity. Appl. Opt..

[24] Takahashi, H. Novel pixel structure with Stacked Deep Photodiode to achieve high NIR sensitivity and high MTF. *2016 IEEE Symposium on VLSI Technology*. IEEE (2016).

[25] Duvernoy, H. M. *The human brain stem and cerebellum: surface*, *structure*, *vascularization*, *and three-dimensional sectional anatomy*, *with MRI*. (Springer Science & Business Media, 2012).

[26] Andrews TJ, Halpern SD, Purves D (1997). Correlated size variations in human visual cortex, lateral geniculate nucleus, and optic tract. J. Neurosci..

[27] Watanabe H (2017). Hemoglobin phase of oxygenation and deoxygenation in early brain development measured using fNIRS. Proc. Natl. Acad. Sci. USA.

[28] Ando, T., Korenaga, T. & Fujii, T. Imaging apparatus including light source that emits pulsed light, image sensor, and control circuit. US10104315B2, United States Patent and Trademark Office, 16 October 2018.

[29] Okada E, Delpy DT (2003). Near-infrared light propagation in an adult head model. I. Modeling of low-level scattering in the cerebrospinal fluid layer. Appl. Opt..

[30] Okada E, Delpy DT (2003). Near-infrared light propagation in an adult head model. II. Effect of superficial tissue thickness on the sensitivity of the near-infrared spectroscopy signal. Appl. Opt..

[31] Funane T, Atsumori H, Kiguchi M, Tanikawa Y, Okada E (2012). Dynamic phantom with two stage-driven absorbers for mimicking hemoglobin changes in superficial and deep tissues. J. Biomed. Opt..

[32] Jacques SL (2013). Optical properties of biological tissues: a review. Phys. Med. Biol..

[33] Simpson CR, Kohl M, Essenpreis M, Cope M (1998). Near-infrared optical properties of *ex vivo* human skin and subcutaneous tissues measured using the Monte Carlo inversion technique. Phys. Med. Biol..

[34] Firbank M, Hiraoka M, Essenpreis M, Delpy DT (1993). Measurement of the optical properties of the skull in the wavelength range 650-950 nm. Phys. Med. Biol..

[35] Van der Zee P, Essenpreis M, Delpy DT (1993). Optical properties of brain tissue. Proc. SPIE.

[36] Kavuri VC, Lin Z-J, Tian F, Liu H (2012). Sparsity enhanced spatial resolution and depth localization in diffuse optical tomography. Biomed. Opt. Express.

[37] Culver JP (2003). Diffuse optical tomography of cerebral blood flow, oxygenation, and metabolism in rat during focal ischemia. J. Cereb. Blood FlowMetab..

[38] Shimokawa T (2012). Hierarchical Bayesian estimation improves depth accuracy and spatial resolution of diffuse optical tomography. Opt. Express.

[39] Boas DA, Dale AM, Franceschini MA (2004). Diffuse optical imaging of brain activation: Approaches to optimizing image sensitivity, resolution, and accuracy. NeuroImage.

[40] Bluestone A, Abdoulaev G, Schmitz C, Barbour R, Hielscher A (2001). Three-dimensional optical tomography of hemodynamics in the human head. Opt. Express.

[41] Olesen PJ, Westerberg H, Klingberg T (2004). Increased prefrontal and parietal activity after training of working memory. Nat. Neurosci..

[42] Tsujimoto S, Yamamoto T, Kawaguchi H, Koizumi H, Sawaguchi T (2004). Prefrontal cortical activation associated with working memory in adults and preschool children: an event-related optical topography study. Cereb. Cortex.

[43] Mayer S (1876). Studien zur physiologie des herzens und blutgeflaesse V. Über spontane blutdruckschwankungen. Akad.Wiss. Wien. Math. Nat.Kl..

[44] Katura T, Tanaka N, Obata A, Sato H, Maki A (2006). Quantitative evaluation of interrelations between spontaneous low-frequency oscillations in cerebral hemodynamics and systemic cardiovascular dynamics. NeuroImage.

