# Supplementary material for: Non-contact acquisition of brain function using a time-extracted compact camera
Source: Sci Rep. 2019 Nov 28;9:17854. doi: 10.1038/s41598-019-54458-7 (PMC6882904; doi:10.1038/s41598-019-54458-7)
Supplement: Supplementary file 1 — Supplementary information [file 41598_2019_54458_MOESM1_ESM.doc]

**Supplementary Materials**

Non-contact acquisition of brain function using a time-extracted compact camera

Takamasa Ando^1*^, Tatsuya Nakamura^1^, Toshiya Fujii^1^, Teruhiro Shiono^1^, Tasuku Nakamura^1^, Masato Suzuki^1^, Naomi Satoi^1^, Kenji Narumi^1^, Hisashi Watanabe^1^, Tsuguhiro Korenaga^1^, Eiji Okada^2^ and Yasunori Inoue^1^

^1^Technology Innovation Division, Panasonic Corporation, Moriguchi, Osaka 570-8501, Japan

^2^Department of Electronics and Electrical Engineering, Keio University, Yokohama, Kanagawa 223-8522, Japan

Correspondence should be addressed to T.A. (ando.takamasa@jp.panasonic.com).

**
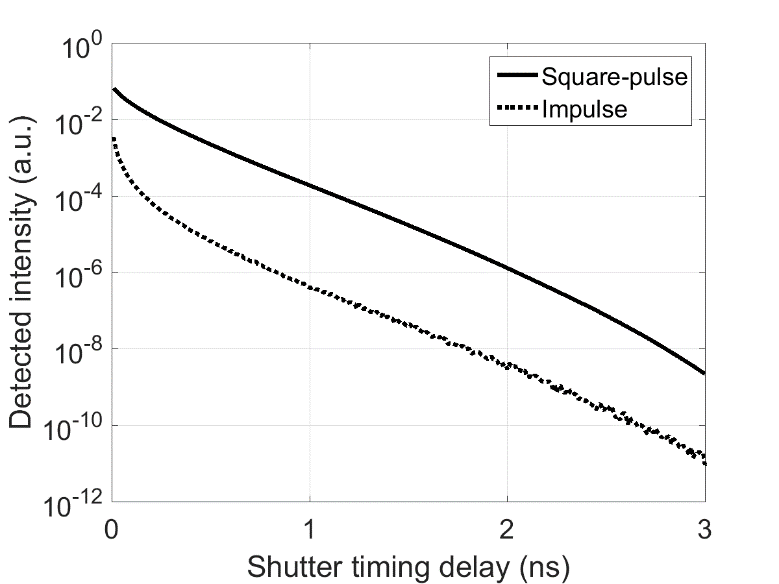
**

**Supplementary Figure S1** | Relationship between the shutter timing delay from the end of the surface reflection component arrival and the signal intensity. These were detected by the sensor using an 11-ns time window. The scattered light in the adult head model was computed using a Monte Carlo simulation with 11 ns square-pulse and 10 ps impulse top-hat pulses as input signals.


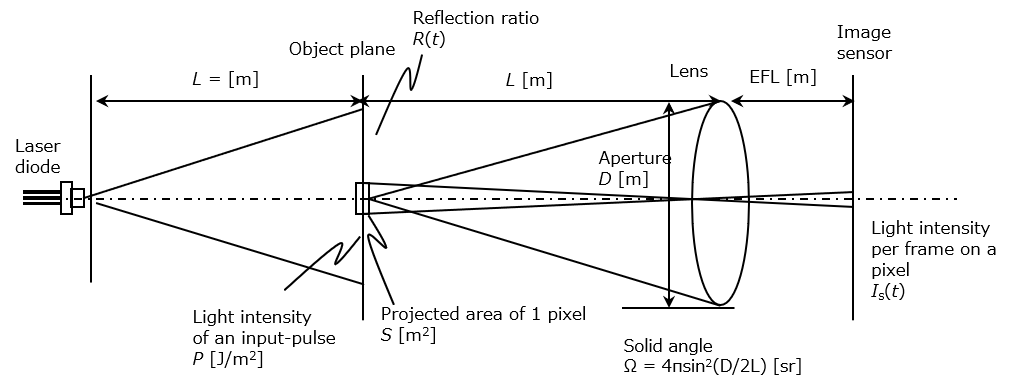


**Supplementary Figure S2** | Parameter for calculating the light intensity on an image sensor.


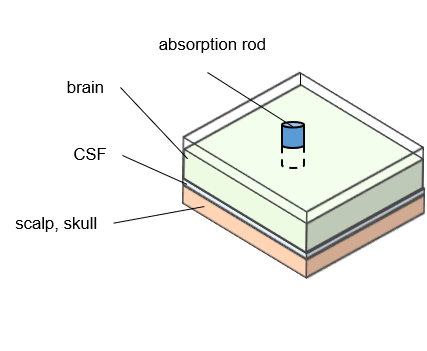


**Supplementary Figure S3 | Head phantom**. This Figure shows the head phantom used in the experiment. The phantom consists of three parts: the superficial tissue (scalp and skull), cerebrospinal fluid (CSF), and brain (Table 1). The outer part of the phantom was constructed of epoxy resin containing TiO_2_ and near-infrared absorbing dye to alter the scattering and absorption coefficients, respectively. The bottom wall consisted of two layers to model the CSF and superficial layers. The phantom consisted of a water bath filled with intralipid solution and green ink to imitate the brain tissue. Two absorption rods were inserted into the intralipid solution to model the absorption changes caused by brain activation. The rods were made of epoxy resin, as was the outer part of the phantom. The diameters of the absorption rods were 10 and 35 mm.

**Supplementary Table S1** The optical properties of each layer

|  | **Thickness (mm)** | **Transport**  **scattering**  **coefficient**  **(mm^–1^)** | **Absorption**  **coefficient**  **(mm^–1^)** |
| --- | --- | --- | --- |
| scalp,  skull | 10 | 1.8 | 0.019 |
| cerebrospinal  fluid | 2 | 0.3 | 0 |
| brain | ∞ | 2.1 | 0.03 |
| absorption rod | ∞ | 2.1 | 0.05 |


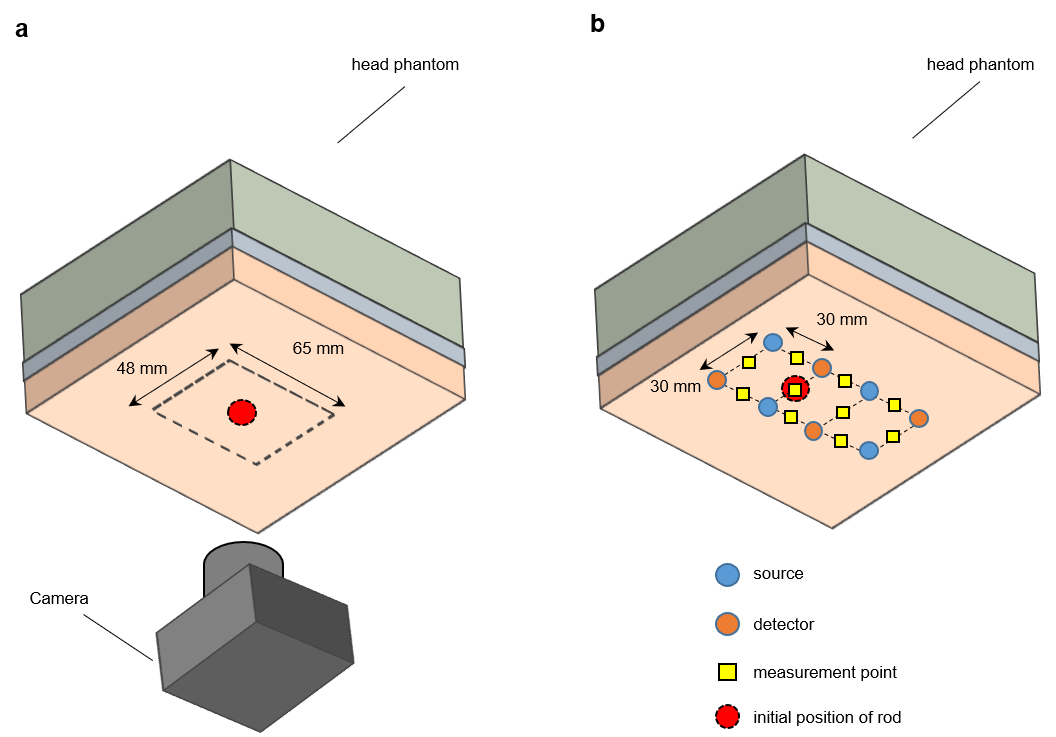


**Supplementary Figure S4 | Measurement area. a**, The proposed camera was set at a distance of 14.5 cm below the head phantom, as shown in **Supplementary Fig. S3**. The camera sensor size is 1/4 inch and the focal length of the lens is 8 mm, which allows the camera to capture an area 48 mm (V) × 65 mm (H) of the target object. **b,** Conventional NIRS were attached to the phantom. Four sources and four detectors were aligned at alternating 30-mm-interval lattice points. The measurement points were defined as the midpoints between the sources and detectors.


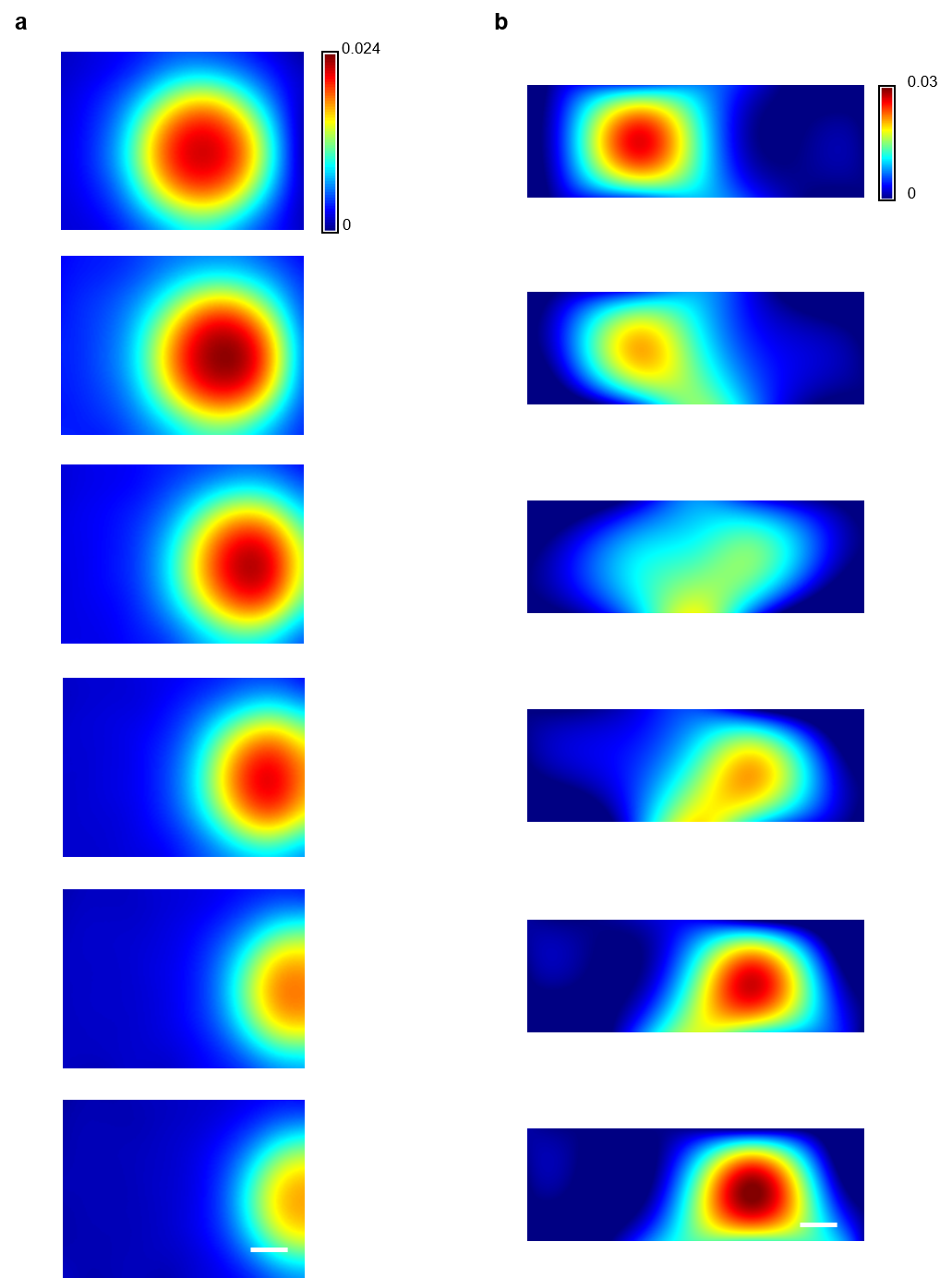


**Supplementary Figure S5** | Detected images of the 35-mm-diameter absorption rod moved horizontally in 6-mm steps through the phantom in **Supplementary Fig. S4**. **a,** **b**, SLICE camera and conventional square sparse NIRS images of the absorption rod, which moved from the centre position shown in **Supplementary Figs. S4a, b**, respectively. The results in **Supplementary Figs. S5a** and **b** showed almost the same size as the absorber with a diameter of 35 mm; however, the image detected by the square sparse NIRS was inaccurate, as shown in the middle of **Supplementary Fig. S5b.** Scale bars, 10 mm.


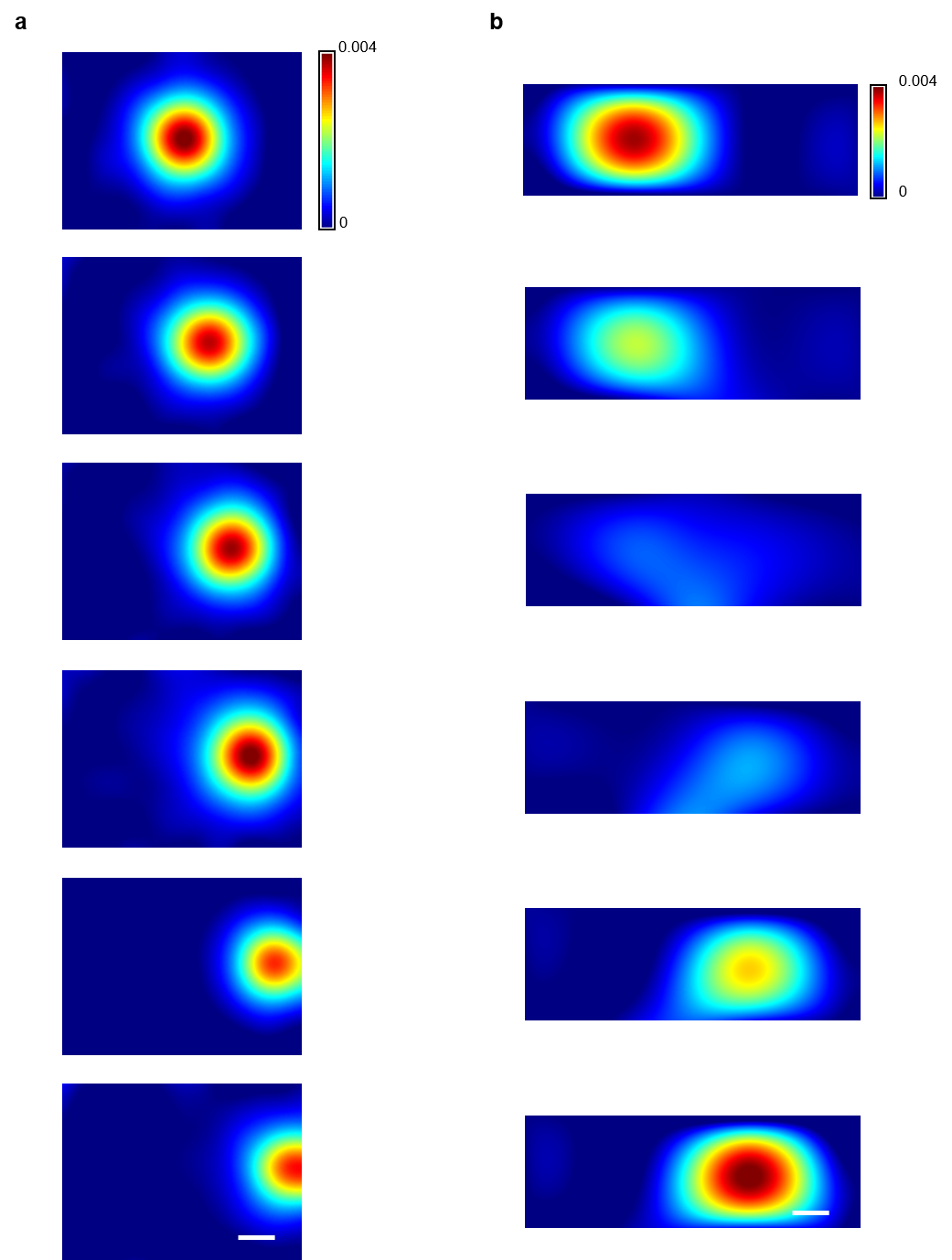


**Supplementary Figure S6** | Detected images of the 10-mm–diameter absorption rod moved horizontally in 6-mm steps, through the phantom in **Supplementary Fig. S4**. **a,** **b**, SLICE camera and conventional square sparse NIRS images of the absorption rod, which was moved from the centre position shown in **Supplementary Fig. S4a, b**, respectively. The process was the same as that in the experiment in **Supplementary Fig. S5.** In the image of the square sparse NIRS when the absorption rod was located between the two measurement points in **Supplementary Fig. S4b**, the signal from absorption rod disappeared as if no absorbers existed, as shown in **Supplementary Fig. S6b**. The detected size of the image acquired by the square sparse NIRS was the same as that for the 35-mm-diameter absorber, except that the deformation or extinction became worse. The image of the absorber acquired by the SLICE camera accurately reflected the difference in size without deformation or interruption as shown in **Supplementary Figs. S6a**. Scale bars, 10 mm.

**Supplementary Video** | Concept video of SLICE camera to explain principle of time-extracted non-contact imaging.
